# Infantile Colic—The Perspective of German and Polish Pediatricians in 2020

**DOI:** 10.3390/ijerph17197011

**Published:** 2020-09-25

**Authors:** Henning Sommermeyer, Hanna Krauss, Zuzanna Chęcińska-Maciejewska, Marcin Pszczola, Jacek Piątek

**Affiliations:** 1 Department of Medicine, The President Stanisław Wojciechowski State University of Applied Sciences in Kalisz, Nowy Šwiat 4, 62-800 Kalisz, Poland; h.sommermeyer@pwsz-kalisz.edu.pl (H.S.); hjk12@poczta.fm (H.K.); 2 Physiology Department, Karol Marcinkowski Medical University in Poznań, ul. Fredry 10, 61-701 Poznań, Poland; zuchecinska@gmail.com; 3 Department of Genetics and Animal Breeding, Faculty of Veterinary Medicine and Animal Science, Poznań University of Life Science, Wolynska 33, 60-637 Poznań, Poland; marcin.pszczola@up.poznan.pl

**Keywords:** crying babies, maternal depression, parental burden, premature termination of breastfeeding, probiotics, shaken baby syndrome, simethicone, synbiotics, early infancy

## Abstract

The objective of the study was to characterize how infantile colic is perceived and managed by German and Polish pediatricians. Data in both countries were collected by using a paper questionnaire with seven questions and predefined and free text fields for the answers. Answers from 160 German and 133 Polish pediatricians were collected. The average of the occurrence rates estimated by both responder groups were at the higher end of published rates. The majority of pediatricians from both countries rated the parental burden caused by infantile colic to be high or very high. Pediatricians’ awareness about the association between infantile colic and maternal depression and premature termination of breastfeeding is relatively well established in both countries. While more than 90% of German pediatricians stated knowledge of infantile colic being a major risk factor for shaken baby syndrome, this knowledge was only declared by half of the Polish responders. Pharmacological interventions, pro-/synbiotics or simethicone, are part of the treatment repertoire of nearly all responding pediatricians. In addition, non-pharmacological interventions (e.g., change of feeding, change of parental behavior) are also among the employed interventions. Results of this study will allow to better design and prioritize communication about infantile colic directed at pediatricians.

## 1. Introduction

Infantile colic is defined by Wessel’s “rule of 3s”, crying or fussing of otherwise healthy newborns for more than three hours per day for more than three days per week for three weeks [1]. Published occurrence rates of infantile colic vary widely, from 3 to 40% depending on details of diagnostic criteria [2]. Infantile colic often begins at around 2 weeks of age, peaks at 6–8 weeks and largely subsides by 3–4 months of age [3]. Although infantile colic is a self-limiting condition, it is a major burden for the baby, the family, health professionals and the health care system. Due to its stressful nature, infantile colic is among the leading causes why parents consult a health care professional during early infancy [4,5].

Infantile colic has been found to be strongly associated with maternal depression, measured with the Edinburgh Postnatal Depression Scale (EPDS) [6]. Longitudinal analyses showed that mothers of infants with colic had increased odds of having high EPDS scores 6 months after delivery even if crying had resolved. While breastfeeding is normally recognized as an effective method of calming infants, it has been reported that mothers of infant colic babies complain that breastfeeding failed to comfort their infants [7]. The same study found that a diagnosis of infantile colic results in significant shorter full breastfeeding duration. Increased crying in otherwise normal infants in the first few months of life has been identified as the strongest risk factor for abusive head trauma (AHT), including shaken baby syndrome (SBS) [8].

Despite many years of research, the etiology of infantile colic remains unclear and many different causes are discussed [5]. Theories range from the assumption that infantile colic is a representation of a severe form of normal infant distress to manifestations of underlying gastrointestinal, neurological or psychosocial disorders. Research in recent years has focused on a link to a disturbed gut microbiota as a potential cause of infantile colic [9]. While the pathophysiological evidence for the role of a disturbed gut microbiota is still not conclusive, there are some important findings supporting this hypothesis. First, a number of studies have found that the gut microbiotas of colicky babies significantly differ from those of non-colicky babies [10]. A lower level of commensal bacteria like *lactobacilli* and *bifidobacteria* and higher numbers of *proteobacteria* were found in the gut microbiota of colicky babies [11,12]. Among these *proteobacteria* were *Escherichia* and *Klebsiella* bacteria [13], well known for their gas-producing properties. Second, it has been described that the gut microbiota of colicky infants exhibits a slower bacterial colonization, a reduced microbiota diversity and a lower microbiota stability [14]. Third, a number of studies has shown that the administration of certain probiotics or synbiotics significantly alleviates infantile colic symptoms, an effect that most likely is related to their effects on the disturbed gut microbiota [15,16,17,18,19]. Fourth, gut and systemic inflammatory markers were significantly increased in colicky babies when compared with controls, which could well be the result of the production of inflammatory lipopolysaccharides (LPS) by *proteobacteria* found in elevated levels [12,20].

Due to the unclear etiology of infantile colic, a large variety of pharmacological and non-pharmacological interventions are employed by pediatricians to address the problem. Simethicone [21], acting on gas bubbles formed in the gut, is frequently used. However, there is no conclusive clinical evidence that simethicone has significant effects in colicky babies [4]. As described above, more promising results have been obtained for treatment with probiotics and synbiotics [15,16,17,18,19]. Non-pharmaceutical interventions comprise changes of parental behavior, feeding adjustments and a number of other approaches (e.g., abdominal massage, osteopathy).

The parental burden caused by infantile colic has been characterized by interviewing affected parents in a study published in 2011 by Landgren and Hallström [22]. To our knowledge, thus far, no study has investigated the pediatricians’ perspective of infantile colic. In the present study, pediatricians in Germany and Poland have been questioned about their assessment of the size of the problem, their understanding of the association between infantile colic and maternal depression, premature termination of breastfeeding and shaken baby syndrome. In addition, pediatricians were asked to elaborate about the treatment approaches they are using in their day-to-day practice to address the problem. Comparing the knowledge about infantile colic of German and Polish pediatricians is of interest as skill shortages, especially in rural areas and smaller towns, provide (Polish and German) physicians who have received their medical education in Poland a work option in Germany.

In Germany, pediatrician education in the area of infantile colic is referencing the S2k guideline 028/041 “Mental disorders in the infant, toddler and preschooler”, published in its latest version in September 2015 [23]. This guideline makes reference to the diagnostic criteria defined by Wessel and provides advice to parents and, if necessary, suggestions for the modification of environmental conditions. Pharmacological intervention for the management of infantile colic is not part of the guideline. Specialized advice centers (in German “Schreiambulanzen”) have been established in Germany, where knowledgeable physicians or midwifes provide advice to parents of colicky babies. In Poland, there is no guideline covering infantile colic, but education of pediatricians is referenced to national [24] and international [25] scientific publications. In contrast to the German S2k guideline, these references are putting more emphasis on the pharmacological treatment options for infantile colic, giving preference to probiotics over surfactants like simethicone.

## 2. Materials and Methods

### 2.1. Surveys

A cross-sectional study which included a survey of German and Polish pediatricians was performed by sending a short cover letter outlining the objectives of the research project and a one-page questionnaire comprising seven questions (Table 1) by regular post to pediatricians in Germany and by e-mail to pediatricians in Poland. Postal addresses for German pediatricians were taken from a commercially available data base. E-mail addresses of pediatricians in Poland were from the private databases of two of the authors (J.P. and H.K., the latter of which is an active member of the Polish Pediatric Society, Polskiego Towarzystwa Pediatrycznego). Pediatricians in Germany were provided with a national fax number to which they were invited to send the completed questionnaire. Pediatricians in Poland scanned the completed questionnaire and sent it back by e-mail. A total of 938 pediatricians in Germany and 372 in Poland were contacted. One reminder was sent to pediatricians who had not responded within two weeks to the first contact. To allow follow-up in Germany, the individual questionnaires were labeled with the name of the answering pediatrician. Data processing was approved by responders by stamp, date and their signature. No incentive of any kind was provided to responders. However, responders could mark a box indicating that they are interested to be informed about the results of the survey.

Answers from returned questionnaires were collected in a database created with the software Excel (Microsoft, Redmond, Washington, DC, USA). Free-text answers were documented in the same database as full text. Keywords in the free text answers were identified and used as basis to analyze this type of answers.

### 2.2. Statistical Analyses

Questionnaires were collected until the data from the last 10 newly collected questionnaires did not change the percentage values of responders of any of the possible (23) predefined answers in the questionnaire by more than 3%. The maximum percentage change caused by the last 10 questionnaires of the German survey was 2.1% and for those of the Polish survey 2.2%.

As a result of quality control of the provided answers, multiple answers given to single choice questions were removed from the datasets. Some of the questions were left with no answers. Such data points were treated as missing values.

The significance of differences between the answers of pediatricians from the two analyzed countries was assessed depending on the nature of the questions. The answer to question (Q) 1 had a form of normally distributed variable, and therefore, the two-sample t-test was used to check for differences in average rates of infantile colic cases. Questions from Q2 to Q4 had ordinal categorical variables, and therefore, for assessing the between-countries differences in the answers an asymptotic Linear-by-Linear Association Test [26] was used as implemented in the coin package [27]. Answers to questions Q5 and Q7 were categorical and non-ordinal, and therefore, the standard Asymptotic Pearson Chi-Squared Test was used. The statistical analyses were performed in R statistical software (R Core Team, Vienna, Austria). In questions Q3 and Q4 the answers “I don’t know” were removed from the dataset during analysis. The significance of differences between pediatricians from Poland and Germany was not assessed for Q6, as this question was a multiple choice question raising analytical difficulties.

## 3. Results

**Figure 1:**
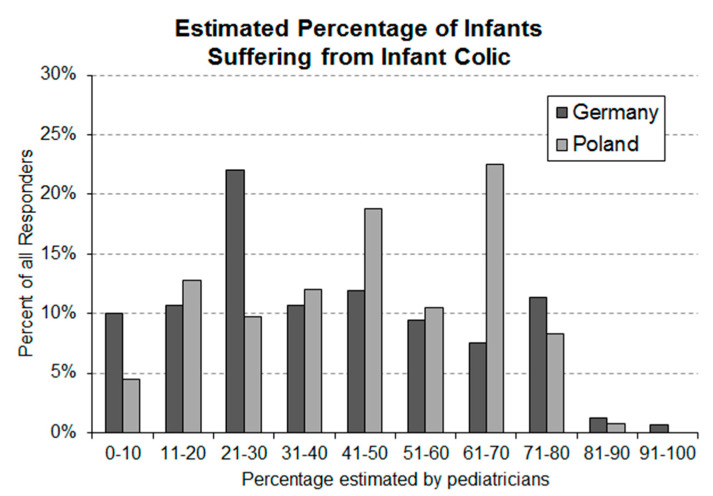
Frequency distribution of German (*n* = 152) and Polish (*n* = 133) pediatricians’ estimates of average occurrence rates of infantile colic in infants aged 0–5 months.

From May to June 2020, responses from 160 pediatricians (responder rate 17.1%) in Germany and 133 (responder rate 35.8%) in Poland were collected. Among the German surveys, there were 11 responses where one question was not answered. In eight of these incomplete questionnaires the estimate for the occurrence rate of infantile colic was missing. The declared average occurrence rate of infantile colic in infants between the age of 0 and 5 months (Q1) in Poland was 49.5% (S.D. 20.8%) and ranged from 10% to 90% (Figure 1). 

### 3.1. Question 1: Estimated Occurance Rate of Infantile Colic in Newborns Aged 0–5 Months

In Germany, the average declared range was 41.6% (S.D. 23.2%) and ranged from 1% to 98%. The two-sample t-test revealed that the difference between the averages was highly significant (*p*-value = 0.002554).

### 3.2. Question 2: Parental Burden Caused by Infantile Colic as Estimated by Pediatricians

**Figure 2:**
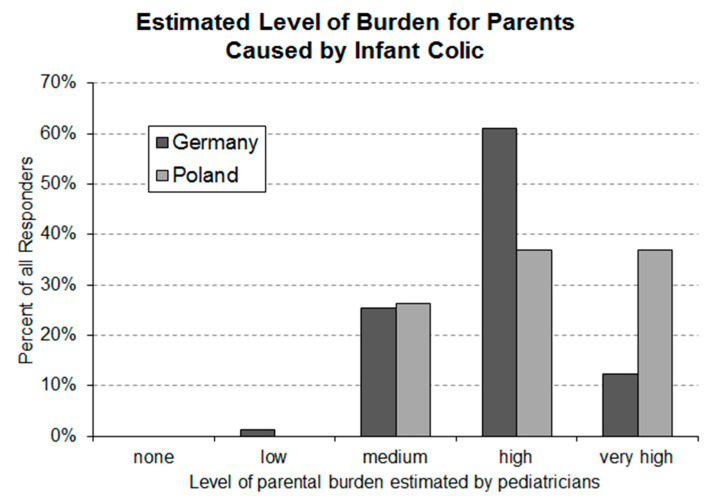
Level of parental burden caused by infantile colic estimated by German (*n* = 159) and Polish (*n* = 133) pediatricians. Statistical analysis using an asymptotic Linear-by-Linear Association Test revealed a highly significant (*p*-value = 0.00255) between-country difference.

Nearly all pediatricians in both countries stated that infantile colic is causing a medium or higher level of parental burden (Figure 2). In total, 61% of the responding German and 37% of the Polish pediatricians assumed that infantile colic causes a high burden for parents. Very high parental burden was assumed by 12% of the German and by 37% of the Polish pediatricians.

### 3.3. Question 3: Estimated Assocoation between Maternal Depression and Infantile Colic

**Figure 3:**
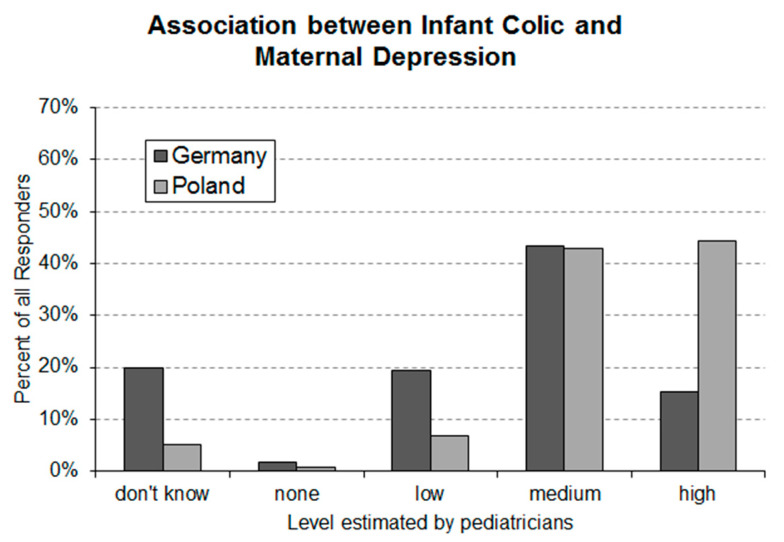
Level of association between infantile colic and maternal depression estimated by German (*n* = 160) and Polish (*n* = 133) pediatricians. The asymptotic Linear-by-Linear Association Test revealed a highly significant (*p*-value = 2.064 × 10^−7^) between-country difference.

In total, 20% of the German and 5% of the Polish responders stated that they do not know about an association between infantile colic and maternal depression (Figure 3). Moreover, 43% of the German pediatricians stated that there is a medium and 15% that there is a high level of association. Of the Polish pediatricians, 42% stated a medium and 44% a high level of association.

### 3.4. Question 4: Estimated Association between Premature Termination of Breastfeeding and Infantile Colic

**Figure 4:**
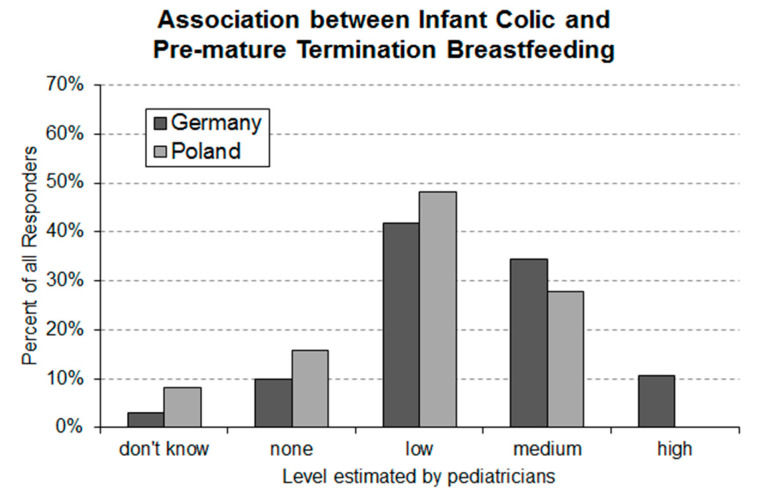
Level of association between infantile colic and premature termination of breastfeeding estimated by German (*n* = 159) and Polish (*n* = 133) pediatricians. An asymptotic Linear-by-Linear Association Test of the data revealed a highly significant (*p*-value = 0.000317) between-country difference.

In total, 3% of the German and 9% of the Polish pediatricians answered that they do not know about an association between infantile colic and premature termination of breastfeeding (Figure 4). A low level of association was assumed by 42% and 49% and a medium level by 34% and 27% of German and Polish pediatricians, respectively. Finally, 11% of German responders stated that they believe that there is a high level of association.

### 3.5. Question 5: Self-Declared Knowledge of Infantile Colic Being a Risk Factor for Shaken Baby Syndrome

**Figure 5:**
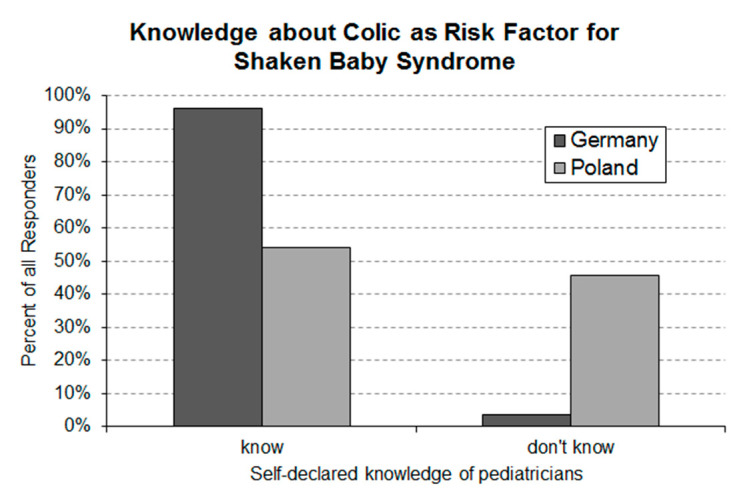
Self-declared knowledge of German (*n* = 159) and Polish (*n* = 133) pediatricians about infantile colic being a risk factor for shaken baby syndrome. Statistical analysis using the standard Asymptotic Pearson Chi-Squared Test revealed a highly significant (*p*-value = 2.2 × 10^−16^) between-country difference.

Of the German responders, 4% declared that they were not aware that infantile colic is a risk factor for shaken baby syndrome (Figure 5). In contrast, 46% of the Polish pediatricians stated that they were not aware of this fact.

### 3.6. Question 6: Interventional Approaches Used by Pediatricians for the Treatment of Infantile Colic

**Figure 6:**
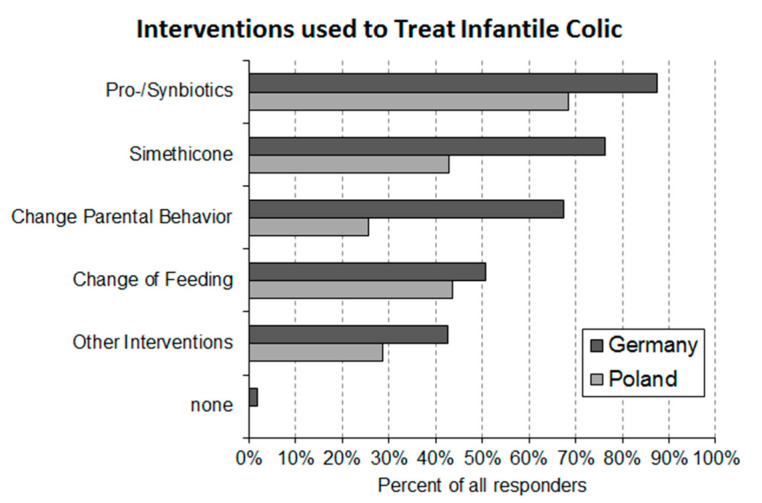
Interventions employed by German (*n* = 160) and Polish (*n* = 133) pediatricians for the treatment of infantile colic.

**Figure 7:**
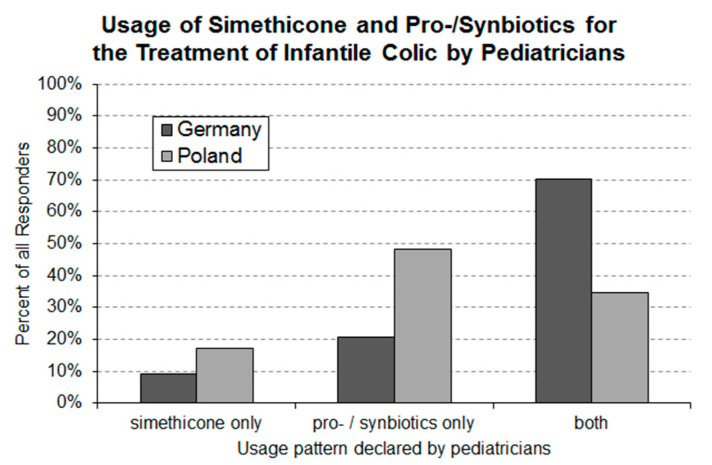
Usage pattern of pro-/synbiotics and simethicone by German (*n* = 160) and Polish (*n* = 133) pediatricians for the treatment of infantile colic. Statistical analysis using the standard Asymptotic Pearson Chi-Squared Test revealed a highly significant (*p*-value = 0.0004724) between-country difference.

Pediatricians are using a variety of approaches to address the problem of infantile colic (Figure 6). The present survey was aiming to investigate what treatment approaches are in the toolbox of pediatricians for the treatment of infantile colic. German responders declared that they are making use of 3.3 ± 1.3 (average ± S.D.) different approaches, while Polish responders employed only 2.1 ± 1.2 (average ± S.D.). For the calculation of these averages the “other interventions” were counted as one treatment. Usage of pharmaceutical products was declared by 97% of the German and by 82% of the Polish pediatricians. The pharmaceutical products employed are either simethicone or belong to the product category of pro-/synbiotics. As under the category “other treatments” hardly any treatments with pharmaceutical products were declared, treatments with pro-/synbiotics and simethicone seem to dominate the pharmaceutical management of infantile colic in the two countries. Pro-/synbiotics are part of the treatment repertoire of 88% of the German and 68% of the Polish pediatricians. Simethicone is employed by a smaller percentage of responders (Germany 77% and Poland 42%). The majority (70%) of German pediatricians stated to make use of both pro-/synbiotics and simethicone (Figure 7) in their daily routine. Of the German pediatricians, 9% declared that they use simethicone exclusively and 21% that they use exclusively pro-/synbiotics as pharmaceutical intervention for the treatment of infantile colic. Of the Polish responders 49% stated to use pro-/synbiotics exclusively, 17% to use only simethicone and 34% to make use of both product types.

Requesting parents to change certain behaviors is popular with 68% of German pediatricians but only with 26% in Poland. In both countries roughly half of the pediatricians suggest changes of feeding (Germany 51% and Poland 44%). In total, 38% of the German pediatricians and 28% of the Polish pediatricians declared that they are using other treatment approaches. In this category treatments such as osteopathy, abdominal massage, teas and administration of products containing cumin can be found (Table 2). None of the responding pediatricians in Poland and only 2% of the German pediatricians declared that they are not employing any treatment at all.

### 3.7. Question 7: Interest in the Topic of Infantile Colic

All responders in Poland and 91% of the German responders stated that they are interested in the topic of infantile colic.

## 4. Discussion

The high occurrence rates and the high parental burden estimated by pediatricians in Germany and Poland confirm that infantile colic is a highly relevant problem in newborns during their first months of life. The average occurrence rates stated in the present study by both responder groups are at the higher end (Germany) or even above (Poland) the rates published [2]. In addition to the high average estimates, the stated individual estimates spread over eight deciles of occurrence rates (Figure 1). There are several potential reasons for this observation. Diagnosis of infantile colic in the daily practice of pediatricians is most likely not based on a strict diagnosis procedure. It is also possible that a large variety of different diagnostic procedures are employed by pediatricians. In addition, the sensitivity of individual pediatricians for the problem of infantile colic might result in a bias that is reflected in the stated estimates of the occurrence rate. In regard to the latter, the found occurrence rates in both countries indicate that a majority of pediatricians are sensitive to the problem, which results in a tendency to overestimate the occurrence of infantile colic. Measures aiming to improve the diagnosis of infantile colic at the level of pediatricians could be helpful to more accurately assess the real size of the problem and to differentiate between infantile colic and elevated, but still normal, levels of crying. Improvements in the diagnosis of infantile colic could influence treatment decisions and support the interaction between pediatricians and parents of colicky babies.

The present study found that the majority of pediatricians is aware of the association of infantile colic and maternal depression. However, with every fifth responder in Germany having stated not to know about this association, future educational programs might consider to elaborate about this association. Over three quarters of responders in both countries stated that the association between infantile colic is high or very high, which is in line with the strong association found by Vik et al. [6].

The recommendation for breastfeeding during the first six months of a newborn’s life [28,29,30] is generally accepted. Knowing the factors that can potentially have a negative impact on breastfeeding is of importance for pediatricians. Over 90% of the responders in Germany and Poland acknowledged their awareness of the association between infantile colic and premature termination of breastfeeding. However, the level of association assumed by the responders is rather medium than high or very high. Motivating mothers of colicky babies to continue breastfeeding, even if it fails to comfort the crying baby [7], is a worthwhile objective to be promoted in front of pediatricians. 

A major finding of the present study is the relatively low awareness of Polish pediatricians about infantile colic being a major risk factor of shaken baby syndrome. While nearly all German pediatricians stated to be aware of this risk, only slightly more than half of the Polish responders declared this knowledge. In recognized cases, shaken baby syndrome typically results in death or extremely damaging injuries. Up to 30% of babies who are shaken and hospitalized die and as many as 70% of survivors suffer long term impairments [31]. While shaken baby syndrome is a rare event, each single case is a tragedy and one case too many. All pediatricians confronted with infantile colic in their daily practice should be aware that colicky babies have an increased risk to become a shaken baby.

The findings of the present study indicate that the majority of pediatricians employ pharmaceutical interventions to manage crying babies. Pharmacological treatment of infantile colic is focused around the intervention with pro-/synbiotics and simethicone. There is increasing evidence that certain pro-/synbiotics improve the crying behavior of colicky babies [15,16,17,18,19]. In contrast, published data for simethicone are more in line with the conclusion that this product is a safe placebo without significant therapeutic effects on infantile colic [4]. Few pediatricians in either country are exclusive users of simethicone. In Poland, nearly 50% of the responding pediatricians exclusively use pro-/synbiotics; the corresponding figure in Germany is slightly above 20%. Moreover, 70% of the German and some 30% of the Polish pediatricians have both product categories in their treatment repertoire. For a significant number of pediatricians in both countries, non-pharmaceutical interventions (e.g., change of parental behavior and change of feeding) are an important part of their treatment repertoire.

The questionnaire employed for data collection of the present study was intentionally kept short so as not to put too much burden on the responders. Therefore, the present study only provides a first and very superficial insight into what treatments pediatricians are currently employing to address the problem of infantile colic in their daily routine. Nevertheless, at least for the pharmacological interventions, it can be concluded that treatments are only partially based on the (limited) available scientific evidence. A future study will have to focus on investigating how pediatricians treat infantile colic in detail. Evidence-based advice how to address the challenges of infantile colic might be helpful to guide pediatricians through the management of colicky babies and their parents.

## 5. Conclusions

Results from the present study provide insights into the current set of believes and treatment approaches of pediatricians in Germany and Poland related to infantile colic, a major problem of infants in the early phase of life. Insights generated by the study will be helpful to guide future communication, training and education programs aimed to improve the management of infantile colic by pediatricians.

## Figures and Tables

**Table 1:** Questions of the survey.

No	Question	Type of Answer
1	What is the average occurrence rate of infantile colic in infants between the age of 0 and 5 months?	Numerical percentage
2	How would you estimate the burden for parents caused by infantile colic?	Selection of one of the pre-defined answers
3	How do you assess the association between maternal depression and infantile colic?	Selection of one of the pre-defined answers *
4	How do you assess the association between premature termination of breastfeeding and infantile colic?	Selection of one of the pre-defined answers *
5	Are you aware that infantile colic is a risk factor for the “shaken baby syndrome” in babies?	Selection of one of the pre-defined answers
6	What kind of treatments do you employ for the management of infantile colic?	Multiple selection of predefined answers and field for free-text answer
7	Are you interested in the topic of infantile colic?	Selection of one of the pre-defined answers

* One of the pre-defined answers was: “I don’t know”.

**Table 2:** Overview of types of other interventions used by pediatricians to address the problem of infantile colic.

Type of Other Intervention	Number of Responders (% of Responders Who Declared the Use of Other Interventions)	
	Germany (*n* = 60)	Poland (*n* = 37)
Change of mother’s diet	None	18 (48.6%)
Abdominal massage	21 (35.0%)	11 (29.7%)
Osteopathy	14 (23.3%)	None
Warm compresses	None	8 (21.6%)
Cumin preparations (incl. Carum carvi *)	12 (20.0%)	none
Homeopathy	7 (11.7%)	none
Tea (fennel, aniseed)	7 (11.7%)	none

* Carum carvi is a homeopathic suppository containing cumin, claiming to have anti-bloating properties.
